# Biomechanical and Biological Assessment of Polyglycelrolsebacate-Coupled Implant with Shape Memory Effect for Treating Osteoporotic Fractures

**DOI:** 10.3390/bioengineering10121413

**Published:** 2023-12-12

**Authors:** Suzy Park, Su-Jeong Lee, Kwang-Min Park, Tae-Gon Jung

**Affiliations:** 1 Medical Device Development Center, Osong Medical Innovation Foundation, 123 Osongsaengmyung-ro, Osong-eub, Heungdeok-gu, Cheongju-si 28160, Chungbuk, Republic of Korea; owner5306@kbiohealth.kr (S.P.); kmpark@kbiohealth.kr (K.-M.P.); 2 R&D Planning Team, Organoid Sciences Co., Ltd., 331, Pangyo-ro, Bundang-gu, Seongnam-si 13488, Gyeonggi-do, Republic of Korea; sujeong12@gmail.com

**Keywords:** poly(glycerol sebacate), biomaterial, osteoporotic fracture, biomechanical, implant

## Abstract

Poly(glycerol sebacate) is a biocompatible elastomer that has gained increasing attention as a potential biomaterial for tissue engineering applications. In particular, PGS is capable of providing shape memory effects and allows for a free form, which can remember the original shape and obtain a temporary shape under melting point and then can recover its original shape at body temperature. Because these properties can easily produce customized shapes, PGS is being coupled with implants to offer improved fixation and maintenance of implants for fractures of osteoporosis bone. Herein, this study fabricated the OP implant with a PGS membrane and investigated the potential of this coupling. Material properties were characterized and compared with various PGS membranes to assess features such as control of curing temperature, curing time, and washing time. Based on the ISO 10993-5 standard, in vitro cell culture studies with C2C12 cells confirmed that the OP implant coupled with PGS membrane showed biocompatibility and biomechanical experiments indicated significantly increased pullout strength and maintenance. It is believed that this multifunctional OP implant will be useful for bone tissue engineering applications.

## 1. Introduction

Bone injuries generated by trauma, accidents, disease, and surgical side effects have become an important issue for clinicians [1]. In addition, with the increase in life expectancy, age-related bone diseases such as osteoporosis and osteoarthritis have become major health issues throughout the world [2,3,4]. According to many reports, 30% of osteoporosis patients are women and the number is increasing every year. This figure reveals that women are about five times more likely than men to suffer from this disease. Furthermore, it is common for both men and women to experience an osteoporotic fracture in the course of their lives. These bone-related diseases pose serious challenges to clinicians and there is a growing urgency to develop advanced treatment strategies [5].

Among the many bone diseases, osteoporosis (OP), which is a systemic metabolic bone disease, is characterized by decreased bone mass and density that is linked with damaged bone microstructure, reduced strength, and increased fracture vulnerability including osteoporotic fracture [6]. Accordingly, as societies rapidly age, the number of patients who suffer from OP is continuously increasing around the world. The traditional treatment strategies for bone diseases include drug management such as injections or medication and surgical treatment of bone tissues, which could produce harmful side effects in other organs, tissues, and neural tubes due to drug toxicity and surgical complications [7]. In particular, surgical treatment utilizing screws, plates, and bone cement is a typical method employed for osteoporotic fracture. However, these types of procedures could damage weakened bone and the surrounding organization because the screw and plate were not fixed in bone [8]. This could require the need for further operations to address outstanding issues. In addition, bone cement, which is additionally used for fixation, may cause leakage that damages other tissues and neural tubes [9].

To overcome these problems, the use of biomaterials such as implants and drug carriers with screws can improve fixation and can also dramatically reduce surgical risks and operation time. Nowadays, studies have suggested various novel construction technologies with modified implants such as new modeling, surface modification, and drug attachment using biomaterials for bone disease therapy [10,11].

Polymeric materials are widely used in the medical and tissue engineering fields [12]. In particular, increasing attention is being paid to biodegradable and biocompatible polymers that have been investigated in relation to restoring tissues and other medical uses (Gadomska-Gajadhur, Wrzecionek, et al., 2018) [13]. Although there are many polymers that can be applied in the field of medicine, it is difficult to achieve satisfactory results as different physical and chemical properties are involved. In this respect, new polymers with improved properties are being continuously experimented with or synthesized to identify appropriate target applications [14,15,16,17].

Shape memory polymers (SMP) are the attracting smart materials that can change shape temporarily and recover to their original shape with temperature. SMPs are increasingly being examined due to their advantageous properties including being light weight and low density, have good flexibility, and their ease of processability [18].

For example, the researchers described how the polycondensation of glycerol with sebacic acid contributes to the obtaining elastomeric biocompatible and biodegradable materials named poly(glycerol sebacate) (PGS) [19]. Poly-glycerol sebacate (PGS) is a polyester elastomer commonly manufactured using the esterification of glycerol with sebacic acid. Additionally, it is a form of degradation that produces non-toxic products [18], because PGS is synthesized without the use of solvents or catalysts. In particular, PGS is confirmed to be bioresorbable in the human physiological environment, through surface erosion during the degradation, which demonstrates slow loss of mechanical strength and thus makes PGS stable after implantation [20,21]. For these reasons, PGS is a very attractive polymer material with a lot of potential applications from soft and hard tissue engineering to drug delivery and sensing devices [19]. In addition, PGS has shape memory properties (SMP), which can remember the original shape and obtain a temporary shape under melting point and then can recover the original shape at body temperature, so it is useful to apply it in the biomedical field due to the fact that customized shapes can be easily obtained [22]. Owing to the unique flexibility of PGS and its malleability, the potential application of PGS in restoring and regenerating soft tissues is particularly noteworthy and exciting [23]. The synthesis of PGS is most commonly produced in two steps, referred to as a cross-linked polymer. The entire process for PGS preparation depends on polycondensation in bulk under a nitrogen gas. Sebacic acid is dissolved in glycerol and then, commonly, is heated to 120–130 °C for 24 h to make PGS prepolymers [24]. This prepolymer can be stored at room temperature as a solution [24]. So, this study chose PGS polymer because it can be manufactured through the curing step in the desired form by using a mold.

**Figure 1:**
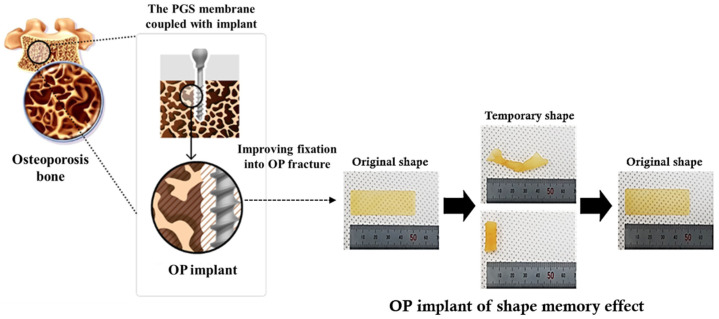
Schematic illustration of the fabrication of the PGS membrane coupled with implant for bone tissue engineering. The OP implant was utilized to improve bone fixation in damaged areas such as OP fractures that are characterized by low bone density and strength to attain successful surgical treatment thanks to the shape memory feature.

Thus, the study discusses how the development of an OP implant with a PGS-coupled implant can be used for bone tissue engineering to improve fixation and prevent surgical side effects including the need for secondary operations in regard to osteoporotic fractures. More specifically, since the drilling process to insert the implant during surgery to treat fractures due to osteoporosis causes damage to weakened bones, the implant is wrapped in the PGS membrane with shape memory characteristics and inserted into the drilling space to recover the shape and improve fixation. In brief, the OP implants were designed to be used to control the curing condition and washing time as depicted in Figure 1 and Table 1. Additionally, in order to completely remove monomers that may remain after curing, a washing step was established and experiments were conducted for each washing condition.

The physicochemical, biological, and biomechanical characteristics of various PGS-coupled implants are described. In vitro cell culture experiments were performed to explore their feasibility for bone disease bioengineering.

## 2. Materials and Methods

### 2.1. Materials

Glycerol and sebacic acid were purchased from Sigma–Aldrich Co., Ltd. (St. Louis, MO, USA). Dulbecco’s modified Eagle’s medium (DMEM), Dulbecco’s phosphate-buffered saline (DPBS), and fetal bovine serum (FBS) were purchased from Gibco (Carlsbad, CA, USA). Deionized–distilled water (DDW) was produced by an ultrapure water system (Puris-Ro800; Bio Lab Tech., Seoul, Republic of Korea). All other reagents and solvents were of analytical grade and were used without further purification. Synthetic bone (sawbone; Pacific Research Laboratory Inc., Vashon Island, WA, USA) was purchased as substitute and it was cut into rectangular blocks with dimensions of 40 mm × 90 mm × 70 mm of open cell rigid foam grade 7.5 pcf (density of 0.12 g/cm^3^; sawbone model #1522-507), which is suitable for the purposes of simulating osteoporotic bone.

### 2.2. Synthesis of Poly(glycerol sebacate) with Various Conditions

A PGS elastomer was fabricated using the conventional method without solvents or catalysts.

**Figure 2:**
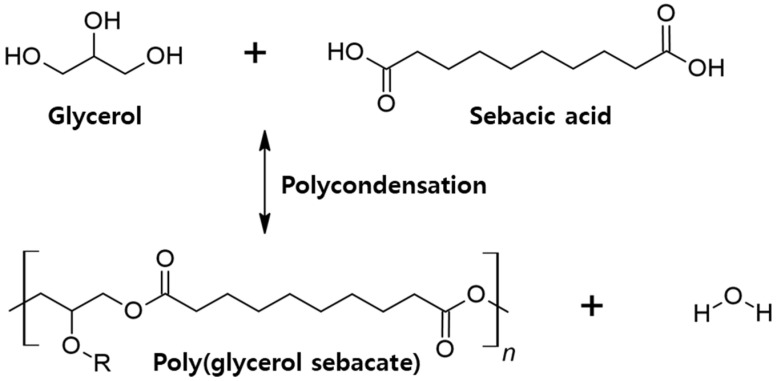
Chemistry of poly(glycerol sebacate) synthesis.

In brief (Figure 2), Equimolar quantities of glycerol (15.64 g, 0.17 mol) and sebacic acid (34.36 g, 0.17 mol) were transferred to a 500 mL three neck round flask. Next, the mixture was maintained at 120 °C under a nitrogen atmosphere for 24 h (prepolymerization conditions). And then, the prepolymers were transferred to a Teflon mold in order to cure and the reaction temperature was maintained at 120 °C, 40 m Torr for 48 h. To achieve the correct conditions for PGS, the process was conducted using a modified method by which various parameters were controlled in relation to PGS prepolymers including the curing temperature, curing time, and washing time as described in Table 1.

Under the washing condition, the cured PGS membrane was put in water and stirred at room temperature.

### 2.3. Characterization

FT-IR analysis was performed in the range 4000–400 cm^−1^ using a spectrophotometer (Spectrum100, Perkin Elmer, Wellesley, MA, USA) by total reflection method. XRD was investigated under 40 kV operating voltage, 40 mA current intensity, and 2θ range from 5 to 45. Thermal transitions were investigated using a DSC (Perkin Elmer Diamond, Shelton, CT, USA) operated at a heating rate of 10 °C min^−1^ under a nitrogen gas flow and the thermograms were collected from −60 °C to 300 °C. The ^1^H-NMR spectrum was measured using a Spectrospin Avance Ultrashield 400 spectrometer (Bruker, Berlin, Germany), operated at 400 MHz. The PGS elastomer samples were dissolved in CDCl_3_ (4 mg mL^−1^) and TMS (tetramethylsilane) was used as a reference [25].

### 2.4. Cell Culture and Experiments

C2C12 mouse muscular cells were cultured in a culture medium containing DMEM supplemented with 10% FBS and penicillin (500 units/mL), and streptomycin (500 units/mL) in a 5% CO_2_ incubator at 37 °C. Cells were maintained in a managed humidified environment and sub-cultured at approximately 80% confluence using trypsin including EDTA, as described earlier [26]. Cell culture was carried out every three days to ensure optimal cell growth and viability. In order to perform the cell culture experiments, specimens extracted causing the decrease below 70% of the cell activity of the control were regarded cytotoxic, as described in the standard ISO 10993-5 [27]. In brief, in order to apply each PGS membrane to cells, 20 mL of physiological saline was added to each 4 g specimen and eluted at 70 ± 2 °C for 24 h. And then, the solution was mixed in the same amount as the DMEM, and then used in the experiment. The control group (Blank) was eluted under the same elution conditions using only physiological saline.

The cytocompatibility of each specimen was measured using a WST-1 cell cytotoxic assay kit (Takara Bio, Otsu, Japan). In brief, WST-1 solution (30 µL) and the free DMEM (300 µL) were mixed and then added to each specimen, with an additional incubation at 37 °C for 30 min. The solutions of each specimen were carried to a 96-well plate and the absorbance at 450 nm was observed by using a microplate reader (Bio-Rad Laboratories, Hercules, CA, USA). This measure allowed for the quantification of cell viability, which also confirmed cell growth in the elution of the PGS membranes. The percentage of cell viability was calculated as follows [27].

(1) $$Cell\;viability\;\left(\%\right)=\left[\frac{\left(A_{s}\right)-\left(A_{b}\right)}{\left(A_{c}\right)-\left(A_{b}\right)}\right]\times 100$$

*A_s_* = Absorbance (absorbance of cells)
*A_b_* = Absorbance of the blank (absorbance of medium and WST-1)
*A_c_* = Absorbance of the control (absorbance of containing cells, medium, and WST-1)

### 2.5. Biomechanical Property Analysis

The screw pullout tests were measured using a universal testing machine (Bionix MTS Acumen^®^ Electrodynamic Test Systems, MN, USA). The test block was inserted with various OP implants as described in Table 1 as outlined in the standard ASTM F543-17 [28]. The ASTM F543-17 test specification is a test method to verify the fixing force between bone tissue and screw nails and measures the load in the tensile direction of the vertical axis when the screw is removed from polyurethane. The results of the fixed force according to this standard are not suitable for direct association with the force required to remove screws from human or animal bones, but the test standard was referenced because it is suitable for measuring the uniformity of the tested specimen and for relative comparison.

Sawbones (Pacific Research Laboratories, Vashon, WA, USA) are standard test materials used to test the biomechanical performance of orthopedic implants. In this study, the cell size that reproduces the bone density similar to that of osteoporosis patients is 1.5–2.5 mm in diameter, with a density of 0.12 g/cc and compressive strength. A polyurethane block with a compressive modulus of 18.6 MPa and 0.28 MPa was used. The fracture combination screw used in the test was a fully threaded design commercial product (OP implant) with a diameter of 4 mm and a fully threaded screw thread with a thickness of 0.5 mm (PGS membrane (0.5 mm)-coupled OP implant) that is most commonly used in fracture combination screws. When inserting a fracture screw into a polyurethane block, a pilot hole with a diameter of 3 mm was implemented in the vertical direction of the test block using a desk drill to minimize the result error caused by the screw insertion angle. After making the pilot hole, the implant was inserted into the test block up to 60% of the total length of the screw line at 3 r/min rotational speed using a screwdriver.

After the screw and the upper jig were aligned in the load axis direction, the test block was fixed using a lower jig. The pullout load was applied under displacement control at a rate of 5 mm/min until the pullout of the OP implants from the test blocks was investigated. Load-displacement data were obtained at a frequency of 30 Hz, and the average and standard deviations were calculated by performing the experiment five times per group.

### 2.6. Statistical Analysis

The results for the biocompatibility test were measured in triplicate and data are expressed as a mean ± standard deviation (SD) unless otherwise noted. This study used a one-way analysis of variance with Tukey’s post hoc comparison to evaluate the statistical significance between the control and various test groups. The analysis was conducted using SPSS statistics software ver. 22 (IBM Corp., Armonk, NY, USA). *p*-values ≤ 0.05 were considered as results with a statistically significant difference compared to the control.

## 3. Results

### 3.1. Produce of OP Implant with PGS

Osteoporosis (OP) implants manufactured by combining a PGS for surgical treatment of osteoporosis fractures often result in crushed fractures. These PGS membranes were expected to tightly fix the implant used for the treatment of fractures in order to address various surgical problems, such as the need for reoperation due to implant loosening, and side effects due to leakage of bone cement into nerve tubes and other tissues. Importantly, the shape memory property of PGS can promote the securing of the fixation of the implant in relation to osteoporosis fractures. PGS ‘remembers’ its initial shape and the temporary shape has to be kept below room temperature, after which the initial shape can be recovered at body temperature due to its low glass transition and melting temperature.

The study further fixed the OP implant coupled with the PGS membrane. After it was inserted it into the drilling area, the initial shape could be recovered and the implant could be tightly fixed into the fracture site. To confirm the effects of fixation and bio-compatibility, the study prepared various PGS membranes such as PGS 1~5, and compared their physicochemical and biological properties.

### 3.2. Physicochemical Properties Characterization

**Figure 3:**
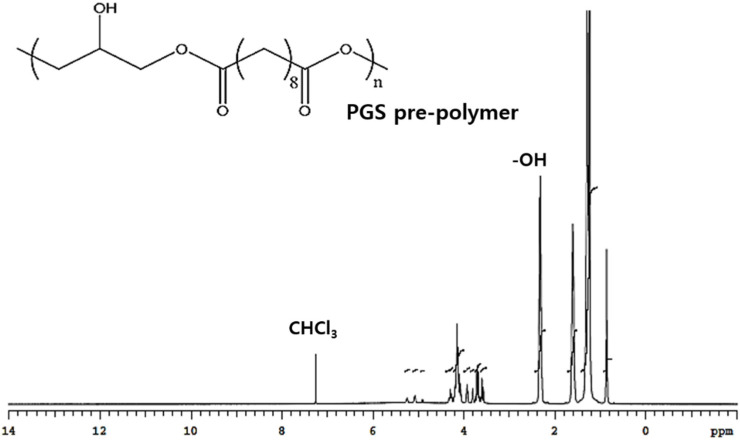
Nuclear magnetic resonance analysis in the spectrum of the PGS prepolymer.

Polymerization was confirmed by nuclear magnetic resonance analysis in the spectrum of the all samples, consistent with successful synthesized PGS polymers (Figure 3). The incorporation of the sebacoyl moiety in the PGS polymers molecular chain were determined from the appearance of the peaks at δ 1.33, 1.72, and 2.25 ppm, respectively [29]. In addition, peaks at δ 3.5–5.5 ppm exhibited in the prepolymer of the PGS spectrum were equivalent to the glyceryl moiety in PGS molecular chain [30]. As shown in the results, the PGS polymers were successfully synthesized.

**Figure 4:**
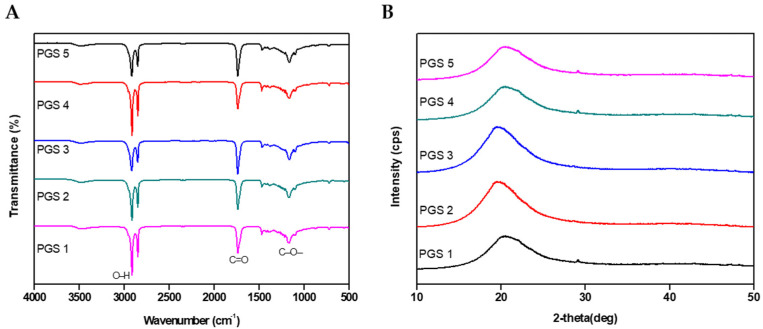
(**A**) Fourier transform infrared spectroscopy of the PGS membranes. (**B**) X-ray diffraction analysis of the PGS membranes.

Fourier transform infrared spectroscopy revealed that various PGS polymers displayed the peak coming from hydroxyl groups of glycerol were exhibited at 3500 cm^−1^ (Figure 4). A peak at 1735 cm^−1^ was expanded and shifted toward higher wave numbers, which means the formation of ester bonds (C=O) took place. The presence of the acid that originated at 1470 cm^−1^ was confirmed and also the intensity of the 1160 cm^−1^ ester band (C-O-) was detected [31,32,33]. In addition, the peak at 1040 cm^−1^ could be ascribed to primary alcohol of glycerol. Based on these results, the successful synthesis of PGS could be confirmed via their compositions, and compared with the chemical structure of PGS shown in Figure 2. From there, the composition of PGS was confirmed.

X-ray diffraction analysis was used to confirm the crystallinity and morphology of the various PGS polymers (Figure 4B). The XRD of PGS polymers exhibited a broad amorphous peak at about 2θ = 20° due to the short range regular ordered structure with free and cured chains in conjunction with the disordered structure of the amorphous phase into the PGS polymers [34].

**Figure 5:**
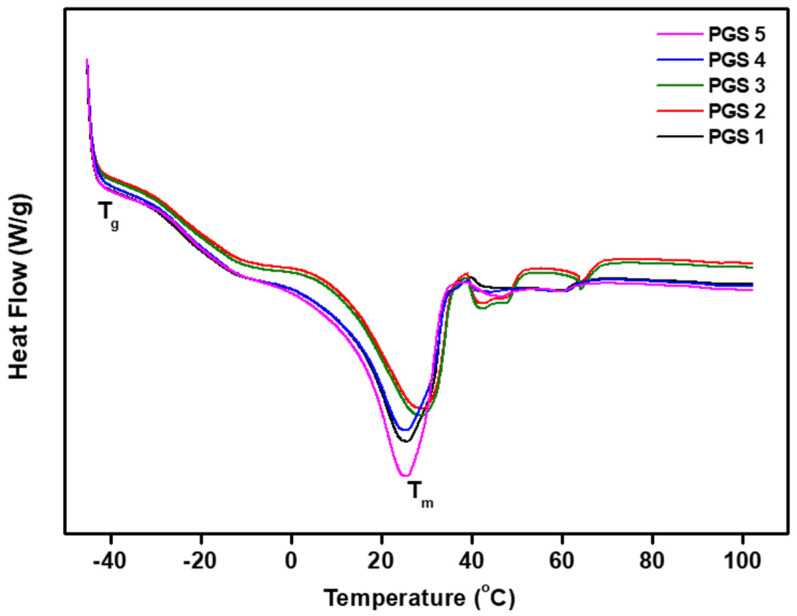
Differential scanning calorimetry of the PGS membranes.

Differential scanning calorimetry was used to observe the thermal properties of the PGS polymers (Figure 5). The PGS polymers have the reversible transitions required for the temporary shape storage of shape memory materials. In addition, these are semi-crystalline polymers, the properties of which depend on the glass transition temperature (T_g_) of the amorphous phase and melting temperature (T_m_) of the crystalline phase. The degree of crystallization is directly related to the extent of the cure. Figure 4 shows that the glass transition of the various PGS polymers ranged between −40 °C and −15 °C with a broad melting transition between −20 °C and −40 °C. This result of the transition is apparent from all the samples produced by various conditions such as synthesis temperature, time, and curing time. Also, the glass transition was investigated with regards to all of them at −30 °C via thermomechanical analysis. Table 2 shows the thermal properties of various PGS membranes obtained from DSC curves at T_g_, T_m_, and T_c_.

**Table 2:** Thermal properties of various PGS membranes obtained from DSC curves.

	T_g_ (°C)	T_c_ (°C)	T_m_ (°C)
PGS 1	−30	−18	25
PGS 2	−29	−13	27
PGS 3	−29	−14	28
PGS 4	−29	−14	25
PGS 5	−30	−15	26

This means that the PGS polymers did not change their structure under the various conditions that were set up and they were maintained in a completely amorphous state at ≥35 °C, which allows the shape memory properties to occur [35,36].

As all of the synthesized PGS membranes showed similar results in chemical analysis such as NMR, FT-IR, XRD, and DSC, it was confirmed that the synthesis conditions did not significantly affect the chemical structure. Therefore, it is thought that there will be no significant difference in the elastomeric properties and shape memory ability of the PGS membrane. In addition, we can hypothesize that the presence of these results can be attributed to the bio-behaviors and biomechanical properties due to a remaining monomer and different degree of crystallization. Therefore, we conducted the experiment focusing on the control of curing to optimize the PGS membrane to apply OP implants for successful bone tissue engineering.

### 3.3. In Vitro Activity and Biomechanical Property Studies

Finally, the feasibility of the PGS membrane coupled implants for OP’s surgical treatment with bioengineering was studied in vitro [27]. As previously stated, as shown in Figure 1, this study reconfirmed whether the PGS membrane had shape memory characteristics even after a five-day washing process after curing, and confirmed that the characteristics of shape memory continued to be maintained.

**Figure 6:**
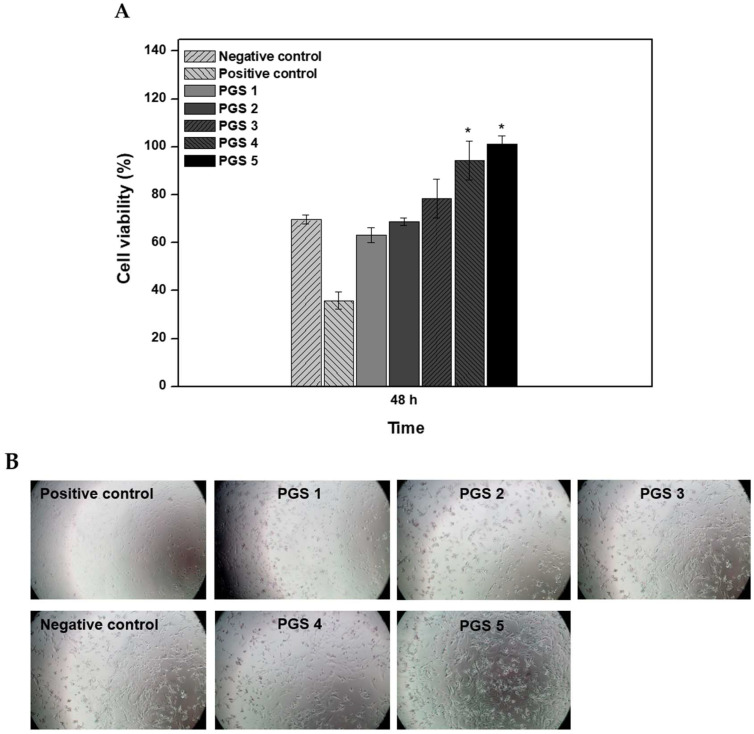
(**A**) Cytocompatibility of C2C12 cell viability on the elution of various PGS membranes. (*n* = 5). We used a one-way SPSS test to evaluate statistical significance between experimental and positive control groups. * The *p*-values ≤ 0.05 were taken as the value with a significant difference as compared with controls. Cell viability on PGS materials was significantly different from the positive controls both PGS 4 and PGS 5. (**B**) Morphological analysis of C2C12 cells grown on the elution of various PGS membranes.

First, the cytotoxicity of cells growing on the elution extracted from various PGS membranes was exhibited in the growth medium by measuring the cytocompatibility and morphological analysis for 48 h (Figure 6). According to ISO 10993-5, the PGS membrane was eluted for 24 h, and then mixed with DMEM medium in the same amount, and this solution was used as a test solution [27]. In the case of the control group, blank, physiological saline excluding the test substance was eluted under the same elution conditions as the test substance, and then DMEM medium was mixed and used. These test solutions were used for testing within 24 h after preparation and were confirmed using the WST-1 measurement method to determine how the eluate of the test substance affects the cells by directly contacting the cells. In addition, the criteria for a standard of judgment of cytotoxicity were used as per ISO 10993-5. By comparing the optical density (OD) value with the OD value of the control group, the cell viability was obtained and when the cell viability was reduced to less than 70% compared to the solvent control group, it was judged to be cytotoxic [26,27].

In this study, the cytocompatibility of cells on each elution of the PGS membranes gradually increased during incubation for all specimens. Most of all, PGS 4 and PGS 5 were determined to have significantly higher cytocompatibility than the other groups. In addition, the conditions of PGS 4 and PGS 5, which were cured for 72 h longer than other groups, were superior to other groups in promoting cell growth. These results mean that PGS 1, 2, and 3 can cause cytotoxicity due to remaining monomers and degraded surface with released acidic components such as the residue of sebacic acid left after failing to bind to glycerol into in vitro [37]. The degradation profile of these PGS materials will be confirmed later through in vivo tests that will create and use a similar environment.

Also, as shown in Figure 6, PGS 4 and PGS 5 were completely cured by the washing method for 5 days into water at room temperature without any acidic components [38,39,40]. As a result of cytocompatibility, it was confirmed that the process of removing the remaining monomers and residues after curing is essential and most of them are removed through a washing process of at least 5 days. Also, based on this result, we confirmed PGS 5, which had the highest cell proliferation, for application with the OP implant using the method of wrapping up the implant with the shape memory effect at human body temperature. Moreover, it was confirmed that the optimized synthesis condition of the PGS membrane to be applied to the OP implant was PGS 5. Therefore, we selected PGS 5 among the five PGS membranes and then combined it with the implant to fabricate an OP implant for the treatment of osteoporosis fractures.

**Figure 7:**
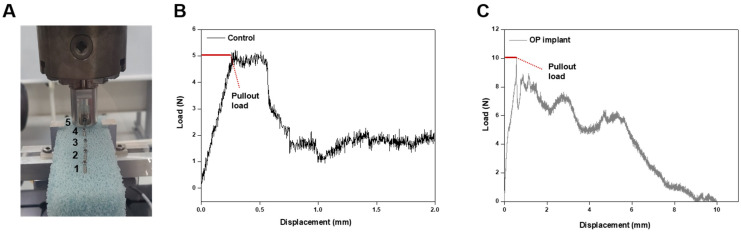
(**A**) The pullout test using MTS 858 testing equipment. (**B**,**C**) The pullout test results of control group and OP implant (*n* = 5).

Next, the biomechanical properties of the PGS membrane (0.5 mm) coupled OP implant with fully thread screw were studied using MTS 858 testing equipment (Figure 7) [41]. According to the pullout test, one of the methods generally used to assess the pullout strength in the case of osteoporosis implant, the strength of the OP implant into the sawbone block was significantly increased compared to the control group as shown in Figure 7. The measured power of the pullout strength of the OP implant (10.06 N) was four times higher than the control group (5.37 N) in Table 3. During the experiment, no breakage or deformation occurred in the screw nails and test blocks. In addition to the calculation of pullout strength of the OP implant, it was increased by 87.4% compared to the control group. It was confirmed that the screw nail for fracture bonding by the shape memory polymer had a pullout prevention effect during a large displacement with a higher load. While the pullout strength of the OP implant had a displacement of 10 mm, the control group displayed a displacement of 2 mm. This means that OP implant had an improved maintenance force into osteoporosis fracture due to the shape memory property [42,43].

In the implant for metal plate fracture combination used for fracture treatment, the metal plate is positioned on the fracture and fixed using a screw nail for fracture combination. However, after surgery, the procedure can fail due to loosening or pulling of the screw as a result of human activity. Moreover, in osteoporosis patients, bone density, strength, and microstructure are all weakened, so surrounding tissues and bones are often broken or damaged in the drilling process required for implant insertion for bone bonding. Accordingly, it was necessary to develop implants used for surgical treatment of fractures caused by osteoporosis. Therefore, in order to secure a strong fixing force with the bone, various research and developments such as screw line design, surface treatment, and fusion medical devices are being actively undertaken. Osteoporosis fractures often lead to pulverized fractures, not simple fractures, and the prognosis for surgery is poor. Therefore, in this study, a method of combining the PGS membrane with the implant screw line was devised based on a general fracture combination implant. In particular, the PGS membrane bonded to the implant has the characteristic of remembering the original shape, temporarily having a temporary shape at a specific temperature, and then returning to the original shape at human body temperature. Using the characteristics of these PGS membranes, the implant was taped into the PGS membrane to temporarily have a shape that is easy to insert into the drilling space. Subsequently, by providing the same environment as the human body temperature, the fixing power of the implant was improved by using the effect of filling the drilling space while returning to the original shape in the drilling space.

Therefore, the OP implant, because it was coupled with the PGS membrane, could be stably retained, and this is thought to have a positive effect on improving fixation in the osteoporosis fracture [44]. For precise determination of the OP implants potential for surgical OP facture treatment, further studies including in vivo transplantation of the OP implants are necessary. According to the results of the biomechanical test, the OP implant, a shape memory polymer fusion implant, showed about five times higher pullout strength and about twice higher maintenance strength compared to the existing general implant. Therefore, the PGS membrane-coupled implant may overcome difficulties with current procedures such as frequent screw loosening and pulling during clinical application. In addition, it is believed that the bone cement used to prevent screw loosening can prevent leakage into nerve pipes and damage to other tissues and prevent various complications.

## 4. Conclusions

Due to the rapid aging of society in recent years, many bone-related diseases, including osteoporosis, are increasing. Osteoporosis has a high risk of fracture because it causes low bone density and low strength, and while surgical treatment is performed in the case of fractures, many problems such as implant loosening and removal and other complications can occur. Accordingly, this study attempted to increase the fixation and maintenance of the implant by coupling the implant with PGS polymer materials that possess shape memory properties in order to promote successful surgical treatment in relation to osteoporosis fractures.

Such OP implants exhibited good biocompatibility and biomechanical properties in various experiments. Especially, in vitro experiments with C2C12 cell culture confirmed the cytocompatibility of the PGS membrane, with cell viability <70% being considered toxic based on the ISO 10993-5 standard. In addition, the pullout test of the OP implant revealed improved fixation into the osteoporosis bone compared with the control group, as evidenced by the higher fixation and maintenance due to their shape memory effect to fill the drilling space.

The study thus demonstrated that the PGS membrane-coupled implant has great potential for applications in bone tissue engineering. Such hybrid implants could also be applied in various fields for effective bone tissue engineering.

## Figures and Tables

**Table 1:** Various conditions of PGS prepolymers including curing temperature, curing time, and washing time.

	Curing Temperature (°C)	Curing Time (h)	Washing Time (Day)
PGS 1	120	48	-
PGS 2	120	48	5
PGS 3	130	48	5
PGS 4	120	72	5
PGS 5	130	72	5

**Table 3:** The pullout strength values of control and OP implant.

	Pullout Load (N)—Max (Aver.)
Control	5.37 ± 1.76
OP implant	10.06 ± 1.94

## Data Availability

The data presented in this study are available on request from the corresponding author.
